# Genome-based exploration of the specialized metabolic capacities of the genus *Rhodococcus*

**DOI:** 10.1186/s12864-017-3966-1

**Published:** 2017-08-09

**Authors:** Ana Ceniceros, Lubbert Dijkhuizen, Mirjan Petrusma, Marnix H. Medema

**Affiliations:** 1 Microbial Physiology, Groningen Biomolecular Sciences and Biotechnology Institute (GBB), University of Groningen, Nijenborgh 7, 9747, AG Groningen, The Netherlands; 2 Bioinformatics Group, Wageningen University, Droevendaalsesteeg 1, 6708 PB Wageningen, The Netherlands

**Keywords:** Specialized metabolism, Natural products, Biosynthetic gene clusters, *Rhodococcus*, *Mycobacterium*

## Abstract

**Background:**

Bacteria of the genus *Rhodococcus* are well known for their ability to degrade a large range of organic compounds. Some rhodococci are free-living, saprophytic bacteria; others are animal and plant pathogens. Recently, several studies have shown that their genomes encode putative pathways for the synthesis of a large number of specialized metabolites that are likely to be involved in microbe-microbe and host-microbe interactions. To systematically explore the specialized metabolic potential of this genus, we here performed a comprehensive analysis of the biosynthetic coding capacity across publicly available rhododoccal genomes, and compared these with those of several *Mycobacterium* strains as well as that of their mutual close relative *Amycolicicoccus subflavus*.

**Results:**

Comparative genomic analysis shows that most predicted biosynthetic gene cluster families in these strains are clade-specific and lack any homology with gene clusters encoding the production of known natural products. Interestingly, many of these clusters appear to encode the biosynthesis of lipopeptides, which may play key roles in the diverse environments were rhodococci thrive, by acting as biosurfactants, pathogenicity factors or antimicrobials. We also identified several gene cluster families that are universally shared among all three genera, which therefore may have a more ‘primary’ role in their physiology. Inactivation of these clusters by mutagenesis might help to generate weaker strains that can be used as live vaccines.

**Conclusions:**

The genus *Rhodococcus* thus provides an interesting target for natural product discovery, in view of its large and mostly uncharacterized biosynthetic repertoire, its relatively fast growth and the availability of effective genetic tools for its genomic modification.

**Electronic supplementary material:**

The online version of this article (doi:10.1186/s12864-017-3966-1) contains supplementary material, which is available to authorized users.

## Background

Specialized metabolites, also known as secondary metabolites, are small molecules that are not essential for growth and reproduction of the producer organism but give them a survival advantage. One example is the production of antibiotics, which inhibit the growth of surrounding organisms competing for the same resources. Specialized metabolites are applied in human society in various ways [1–4] and comprise diverse classes of chemicals, including polyketides, peptides (produced either ribosomally or nonribosomally), saccharides, terpenes and alkaloids [5].

The bacterial genus that has been most extensively studied for its capacity to produce such bioactive compounds is *Streptomyces*. Streptomycetes are the source of most of the natural antibiotics that are used in modern medicine [6]. Antibiotics were a revolution in medicine, being the cure for many—until then—deadly illnesses such as the plague, leprosy, tuberculosis or syphilis. Unfortunately, many pathogenic bacteria have developed resistance to antibiotics, in some cases even to all antibiotics currently available [7]. Several large-scale efforts are under way to find new antibiotic compounds that can be used to fight these strains. However, in many cases, these efforts suffer from frequent rediscovery of compounds previously identified from other strains [8]. The development of bioinformatics tools to analyse the growing amount of available bacterial genomic sequence information has shown that the number of biosynthetic gene clusters (BGCs) that may encode pathways capable of producing specialized metabolites is much greater than initially thought, even in strains that were already known for their specialized metabolite repertoires [9, 10], but also in many other strains from a wide range of taxonomic groups [11, 12].

One of the actinobacterial genera that has received relatively little attention from the natural products research community is *Rhodococcus*. Rhodococci are actinomycetes that contain mycolic acids in their cell walls; they are closely related to the genus *Mycobacterium*, host of hazardous pathogenic strains such as *Mycobacterium tuberculosis* and *Mycobacterium leprae*. Two *Rhodococcus* species—*Rhodococcus equi* and *Rhodococcus fascians*—are animal and plant pathogens, respectively. Traditionally, *Rhodococcus* strains have been studied for their capacity to degrade complex organic compounds and many of them have been isolated from chemically contaminated environments [13, 14]. A recent study has shown that rhodococci not only have a vast specialized catabolic repertoire, but also a large specialized anabolic repertoire: the genomes of four *Rhodococcus* strains were shown to harbour a vast number of different BGCs, including a strikingly high number of nonribosomal peptide synthetase (NRPS)-encoding BGCs compared to other actinobacteria [12]. NRPSs are modular enzymes, in which each module adds a specific monomer to the peptide backbone. NRPS modules contain at least an adenylation domain (A) that determines the specificity for the substrate and activates it to start the synthesis, a thiolation (T) or peptide carrier protein (PCP) domain that binds covalently to the activated residue and a condensation domain (C) that elongates the peptidyl chain. Different types of C domains catalyze different types of condensation reactions [15]. Some NRPS assembly lines start with a special condensation domain, the C-Starter domain, which catalyzes the acylation of the first residue of the peptidyl chain, thereby synthetizing a lipopeptide [15]. The last module of the NRPS usually contains a thioesterase module that releases the final peptide chain [16].

Among the specialized metabolites previously described in *Rhodococcus*, there are several siderophores: the hydroxamate-type siderophores rhequichelin, heterobactin, rhodochelin [17–19], and the catecholate-type siderophore rhequibactin [20]. Additionally, multiple *Rhodococcus* strains have also been reported to produce antibiotics [21]. Four (groups of) rhododoccal natural products with antimicrobial activity have been described in literature; lariantin peptide antibiotics with anti-mycobacterial activity [22], the polyketide aurachin RE from *Rhodococcus erythropolis* JCM 6824 (which has a structure similar to that of aurachin C from the Gram-negative organism *Stigmatella aurantiaca* [23, 24]), a group of peptide antifungals named rhodopeptins [25] and the recently described humimycins [26]. The gene cluster responsible for the synthesis of rhodopeptins has not yet been identified.

Here, we performed an extensive genomic analysis of the biosynthetic potential of twenty *Rhodococcus* strains with complete genome sequences available. In view of the close phylogenetic distance that *Rhodococcus* has with *Mycobacterium*, we also analyzed several *Mycobacterium* strains: four free-living strains and three obligate pathogens. Also the only available complete genome sequence from the newly discovered genus *Amycolicicoccus* [27], which is a taxonomic relative of *Rhodococcus* and *Mycobacterium*, was included, adding to a total of 28 strains. Based on a computational reconstruction of gene cluster families (GCFs) in these strains, we found several BGCs shared between all species that may play key roles in survival in pathogenic strains and could therefore be studied as potential drug targets, as well as clade-specific clusters that have a high probability of synthesizing novel natural products not previously described. Particularly, a striking variety of putative lipopeptide BGCs was observed. Most of the NRPSs in *Rhodococcus* strains contain an N-terminal condensation domain that belongs to the C-starter subfamily,; additionally, we found a range of different CoA-ligases encoded in NRPS BGCs that may be involved in lipidation. Altogether, we provide a comprehensive overview of the genomic basis of *Rhodococcus* specialized metabolic diversity and show that *Rhodococcus* is a promising and thus far underexplored target genus for natural product discovery, in view of the large number of unknown clusters present in their genomes and the availability of techniques for genetic manipulation of this genus [28].

## Results and discussion

### *Rhodococcus* has great potential for specialized metabolite production

In order to establish a phylogenetic framework to understand biosynthetic diversity in rhodococci and their relatives, a 16S rRNA phylogenetic analysis was first performed with all 28 selected strains and two *Streptomyces* species as outgroups: the model organism *Streptomyces coelicolor* and the industrial clavulanic acid producer *Streptomyces clavuligerus,* both of which are well-known specialized metabolite producers (Additional file 1: Figure S1). The tree shows a structure that corresponds to the previously described paraphyletic nature of the *Rhodococcus* genus [29, 30].

**Fig. 1:**
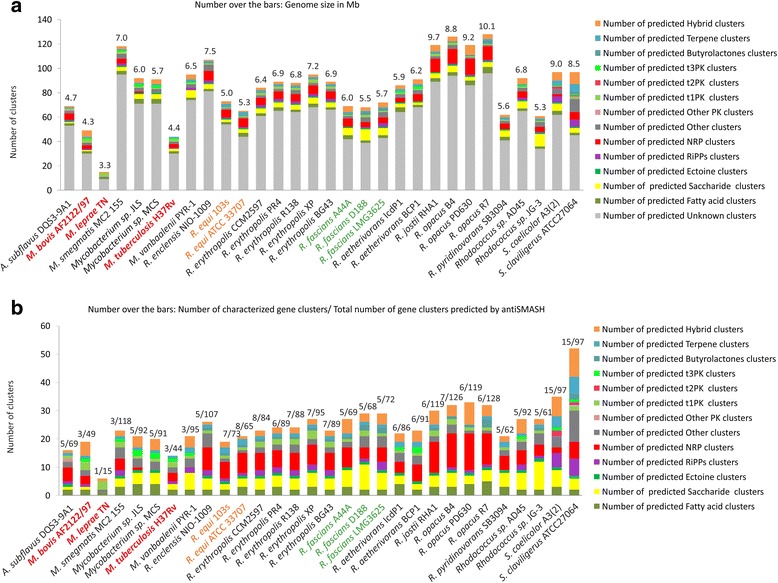
Numbers and families of biosynthetic gene clusters (BGCs) detected in all studied strains, compared to the well-known producers *S. coelicolor* and *S. clavuligerus*. Plant pathogens are marked in green, animal and opportunistic pathogens in orange and human pathogens in red. **a** Total number of BGCs predicted by antiSMASH, including ClusterFinder. On top of each bar, numbers represent the size of each genome in Mb. **b** BGCs predicted by antiSMASH to code for known families of biosynthetic pathways. On top of each bar, proportion of described gene clusters from each strain. It can be observed that the proportion of NRPS BGCs in *Rhodococcus* compared to other types of molecules is much higher, especially in *R. jostii* RHA1 and *R. opacus* strains. (T3PK: Type III polyketide. T2PK: Type II polyketide, T1PK = Type I polyketide, PK: Polyketide. NRP: nonribosomal peptide. RiPP: ribosomally synthetized and posttranslationally modified peptide)

A computational analysis (using antiSMASH 3.0 + ClusterFinder [11, 31, 32]) of their biosynthetic capacity showed a considerable number of BGCs in all 28 strains (Fig. 1, Additional file 1 and Additional file 2). antiSMASH predicts gene clusters that belong to known biosynthetic classes, while ClusterFinder searches for putative gene clusters of unknown function based on observed local protein domain frequencies [11, 33]. The strain with the highest number of gene clusters (128 BGCs, out of which 32 belong to known families and 96 are putative ClusterFinder-predicted BGCs) is *Rhodococcus opacus* R7, which also has the largest genome with 10.1 Mb; it is followed by *Rhodococcus jostii* RHA1 and the other *R. opacus* strains. The strain with the fewest gene clusters (*M. leprae*, with 15 BGCs, among which 9 are ClusterFinder-predicted putative BGCs) has the smallest genome with 3.3 Mb (Fig. 1a). Both in *Mycobacterium* and *Rhodococcus*, pathogenic strains generally have a smaller genome and a limited array of BGCs [34]. Obligate pathogens live inside a host, reducing the need to adapt to sudden environmental changes; also, they probably encounter a smaller diversity of microbial competitors in their environment compared to soil bacteria, which may reduce the need of different biosynthetic and/or catabolic pathways could contribute to genome minimization [35]. They still need a minimum arsenal of molecules to compete for resources with the host they infect. For instance, *M. tuberculosis* is known to require siderophores to capture iron, which is in most cases in low availability since the host organism sequesters it for its own use [18, 20, 36]. These siderophores are therefore targets to treat mycobacterial infections.

Interestingly, the percentage of ClusterFinder-predicted putative clusters that are not assignable to a known type of molecule is about 75% of the predicted BGCs in *Rhodococcus*, compared to the 64% and 46% for *S. coelicolor* and *S. clavuligerus* respectively (Fig. 1b). These putative BGCs contain diverse types of enzyme-coding genes, different between each cluster. While ClusterFinder-predicted BGCs have an inherent high uncertainty regarding their biochemical function (which in some cases can also be non-biosynthetic), this does indicate that these genomes may encode unknown pathways for the biosynthesis or modification of a large variety of thus far unknown molecules.

Altogether, the above findings corroborate the study of Doroghazi and collaborators [12], who have recently studied a smaller number of rhodococcal and mycobacterial genomes; they noted that these genomes contain a large number of putative BGCs, and that the NRPS vs PKS (Polyketide synthase) ratio in rhodococci is higher compared to that in other actinomycetes*.* Our results show that these observations extend throughout the *Rhodococcus* genus (Fig. 1). Indeed, most of the *Rhodococcus* strains contain a range of NRPS-encoding gene clusters but only one or two Type I PKS-encoding gene clusters, and they do not contain any Type II or Type III PKS clusters, except for the two strains of *Rhodococcus aethrivorans* and *Rhodococcu*s sp. AD45 that contain one or two Type III PKSs.

**Fig. 2:**
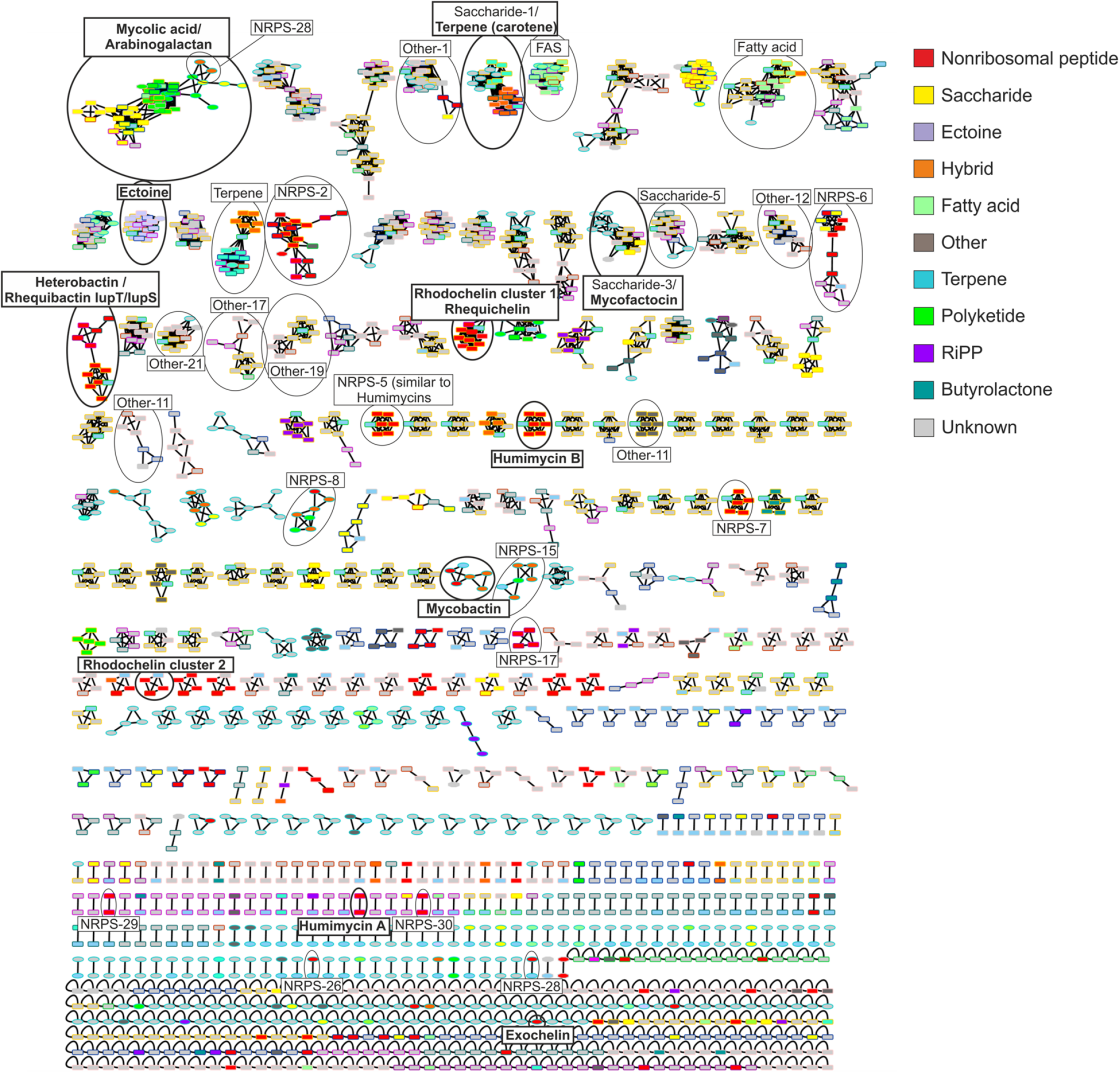
Sequence similarity network relating the gene clusters detected by antiSMASH from all strains. Each cluster is represented by a square in the case of *Rhodococcus*, as an ellipse in the case of *Mycobacterium*, or as an octagon in the case of *Amycolicicoccus subflavus*. The different shapes are colour-coded for different BGC families with a colour scheme consistently applied in other figures as well. The upper part of the network represents the group of GCFs shared across multiple strains; GCFs in the top row are present in all strains and therefore might be crucial for survival. The lower part contains the clusters that are only present in a few or even only a single strain and that could lead to the production of novel compounds that have not been found in other species. GCFs with known product and GCFs discussed in this work are marked with a circle and named

One aim of this analysis was to identify highly conserved biosynthetic pathways with an increased likelihood of being essential to rhodococcal and mycobacterial metabolism and would thus present possible drug targets to combat pathogenic strains; a second aim was to identify gene clusters that are only present in one or a few strains, which may encode biosynthetic pathways for the production of novel bioactive compounds. For this purpose, we used the BiG-SCAPE software (Navarro-Muñoz, Yeong, Medema et al., in preparation) to construct a sequence similarity network that categorizes the different GCFs, thus providing a powerful visualization of shared and strain-specific BGCs across all the studied strains (Fig. 2 and Additional file 3).

In order to more fully understand the evolutionary histories of all studied strains that have led to the currently observed BGC repertoires represented in the network (Fig. 2), we used ancestral state reconstruction with Count [37] to identify the most parsimonious BGC gain/loss events (Additional file 1: Figure S2). For that analysis, we used a total of 114 different BGCs: all families shared between more than 7 strains, complemented by all remaining NRPS BGCs (Additional file 2). Additional file 1: Figure S2 shows which GCFs are conserved across lineages and which ones are not. Twenty-four GCFs are jointly present in the genomes of different strains of *Mycobacterium* and *Amycolicicoccus* (which form a monophyletic clade), as well as in *Rhodococcus*. Altogether, all *Rhodococcus* clades share 36 GCFs, the previous 24 plus 12 more GCFs that are only present in all *Rhodococcus* strains. Notably, it can be observed that the branches leading to *R. jostii* RHA1 and the three strains from *R. opacus* show many putative GCF gain events (Additional file 1: Figure S2), which indicates that ecological specialization of these strains involved acquisition of several biosynthetic pathways through horizontal gene transfer.

### Shared GCFs may have essential functions and therefore offer possible targets to combat pathogenic strains

**Fig. 3:**
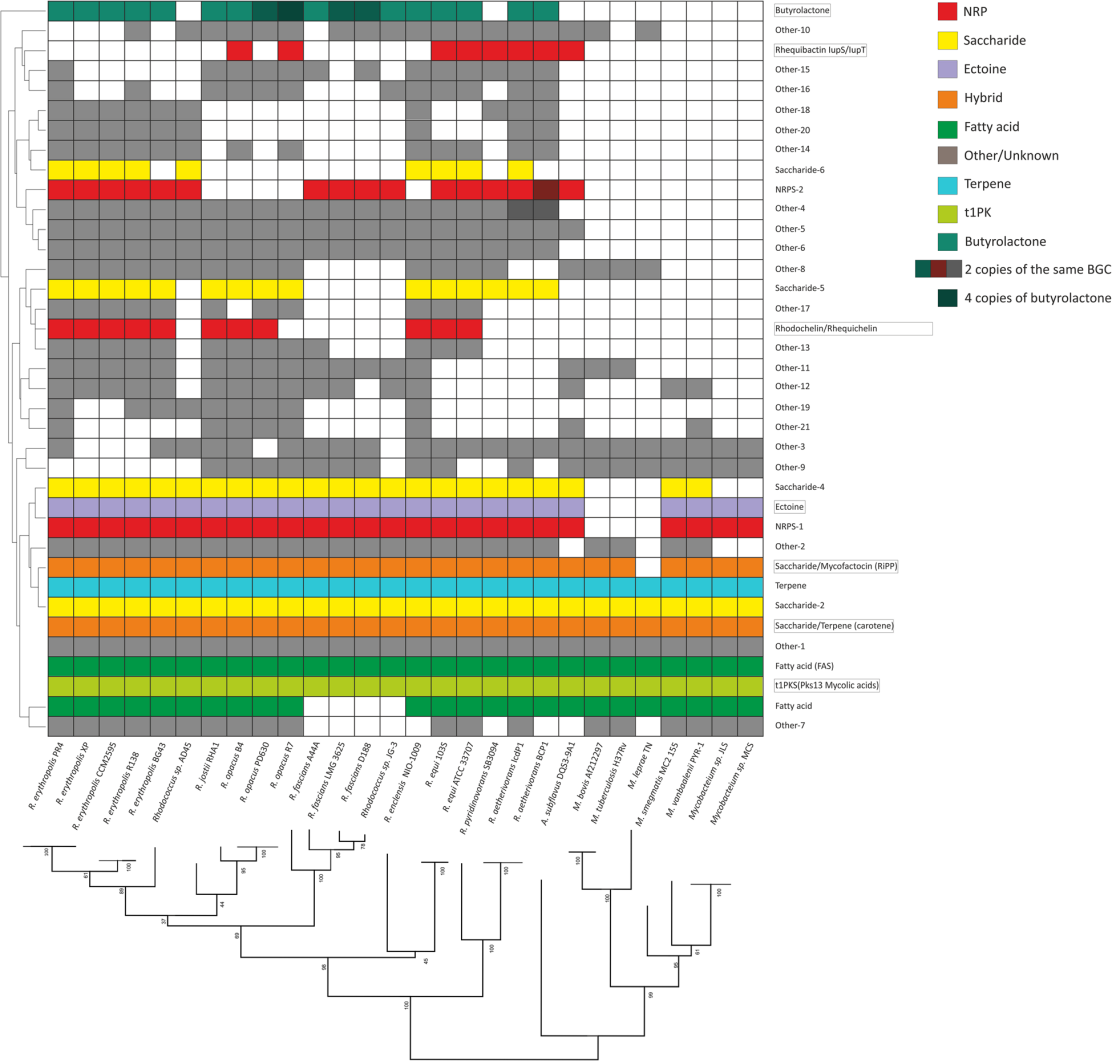
Presence/absence map representing the GCFs shared between more than seven strains. The strains are displayed horizontally in phylogenetic order. Vertically, they were ordered using hierarchical clustering (see Methods for details). Coloured squares indicate the presence of the gene cluster in a specific strain. Darker areas represent more than one copy of the cluster. Six GCFs are conserved across all genomes. Characterized clusters are marked by a square

A detailed analysis was performed on the GCFs shared among more than seven strains, which amount to a total of 37 GCFs. We reasoned that biosynthetic pathways strongly conserved between *Mycobacterium* and *Rhodococcus* may offer possible drug targets in pathogenic strains from both genera, since their conservation suggests that they are important for survival. A presence/absence map of these 37 GCFs in each strain was constructed to provide an overview of these data (Fig. 3).

The vast majority of these GCFs have no experimentally characterized members. Seven GCFs have members that have been previously described in at least one strain: 1) the Type I PKS cluster that contains the gene *pks13* catalyzes the last condensation step of **mycolic acid** biosynthesis [38], which is present in all studied strains. This cluster is grouped together in the network with a saccharide cluster that may also be related to cell wall biosynthesis: it encodes arabinogalactan biosynthetic enzymes, family 2 glycosyltransferases and O-antigen transporters; 2) the NRPS BGC encoding the biosynthesis of the siderophore **rhodochelin and rhequichelin**, present only in 11 *Rhodococcus* strains [39]; 3) the **carotenoid** BGC [40], detected as “Unknown” in *Mycobacterium* strains and *Rhodococcus sp*. AD45; 4) the **mycofactocin** BGC, encoding the biosynthesis of a ribosomally synthesized and post-translationally modified peptide of unknown function that was initially discovered by bioinformatics analysis [41, 42], and is only absent in *M. leprae*; 5) the heterobactin gene cluster described in *R. erythropolis* PR4 and present only in *R. erythropolis* strains, *R. enclensis* and *R. sp.* AD45; 6) the **butyrolactone** gene cluster, detected by the presence of an *afsA* homologue, encoding the main biosynthetic enzyme of the γ-butyrolactone signalling molecules known to be involved in the regulation of the secondary metabolism in *Streptomyces* [43], which are present in all *Rhodococcus* strains except for *Rhodococcus* sp. AD45 and *R. pyridinovorans* SB309; 7) the NRPS encoding the biosynthesis of the siderophore rhequibactin, only present in *R. equi* strains [20].

Five of the 37 GCFs are present in all strains from the three genera studied; each of them is predicted to encode a biosynthetic pathway for molecules belonging to a different family. One of them is the already mentioned **Type 1 PKS** ***pks13***, involved in mycolic acid biosynthesis [38]. All these organisms are known to possess mycolic acids in their cell walls, including *A. subflavus,* but these mycolic acids vary in their complexity [38, 44]. The GCF called **Terpene,** which is also shared by all strains, includes a lycopene cyclase as well as genes encoding the enzymes SufD and NifU, known to be involved in the biosynthesis of iron-sulphur clusters, which are known to be cofactors of various proteins [45]. The third universally shared GCF is the **Saccharide-2** family; BGCs that are members of this family contain genes for the synthesis of menaquinone synthesis, which is also known as vitamin K2 and, among other functions, plays a role in the respiratory electron transport chains in bacteria. It also is known to be an important factor in the latent phase of infection in *M. tuberculosis* [46]. Two other GCFs are shared between all species, for each of which the function is less certain. One of them is predicted to contain fatty acid BGCs encoding a **fatty acid synthase (FAS)**; the other one (**Other-1**) contains BGCs that may be involved in heme biosynthesis. Characterizing the function and products of these clusters may lead to the identification of novel targets to develop vaccines against pathogenic strains such as *M. tuberculosis, Mycobacterium bovis, M. leprae*, *R. fascians* or *R. equi*.

Another GCF present in all strains encodes predicted saccharide-terpene hybrid clusters (indicated as **Saccharide/terpene (carotene)** from now on). However, a closer analysis shows that these ‘hybrid’ clusters probably constitute pairs of unrelated clusters that are adjacent on the chromosome and are therefore (probably incorrectly) assigned as ‘hybrid’ clusters by antiSMASH. Indeed, some GCF members represent just the terpene cluster or just the saccharide cluster.

Among the partially shared GCFs, it is worth mentioning that the **Ectoine** GCF and the NRPS-1 GCF are present in all strains except for pathogenic mycobacteria. Ectoine is an osmolyte that is produced in high salt conditions as osmoprotectant [47]. This molecule therefore does not seem essential for these three pathogenic *Mycobacterium* strains as osmoprotectant. Besides an NRPS, the **NRPS-1** BGCs encode a probable multidrug resistance transporter from the MFS family; the presence of the transporter-encoding gene indicates that this gene cluster may encode the biosynthetic pathway of a bioactive molecule for which self-resistance is needed. The NRPS has one module which contains an adenylation domain without a clear substrate prediction and a peptidyl carrier protein domain. Three other clusters of unknown function (Other-4, Other-5 and Other-6) were detected in all *Rhodococcus* strains, for which we were unable to predict a function. A more detailed description of these gene clusters can be found in Supplementary Information 2 (Additional file 1).

### *Rhodococcus* sp. AD45 and *R. pyridinovorans* SB3094 show a possible high adaptation of their secondary metabolism to their environment

Figure 3 shows that *Rhodococcus sp*. AD45 lacks nine clusters from GCFs that have members in all its close relatives: the **Saccharide-5, Other-11, 12, 13, 17, 19, 21, and** the **Butyrolactone** and the **Rhequichelin/Rhodochelin** gene clusters. This strain was isolated from fresh water sediments and is able to use isoprene as sole source of carbon [48]. However, its genome is not much smaller than those of its closest relatives, the *R. erythropolis* strains. Indeed, this is the third *Rhodococcus* strain for which the majority of its BGCs belong to species-specific GCFs (as will be further discussed later in this work), which suggests that this strain has adapted to a very specific environment by losing some otherwise conserved gene clusters, and by gaining others, probably through horizontal gene transfer from other strains in its environment.

**Fig. 4:**
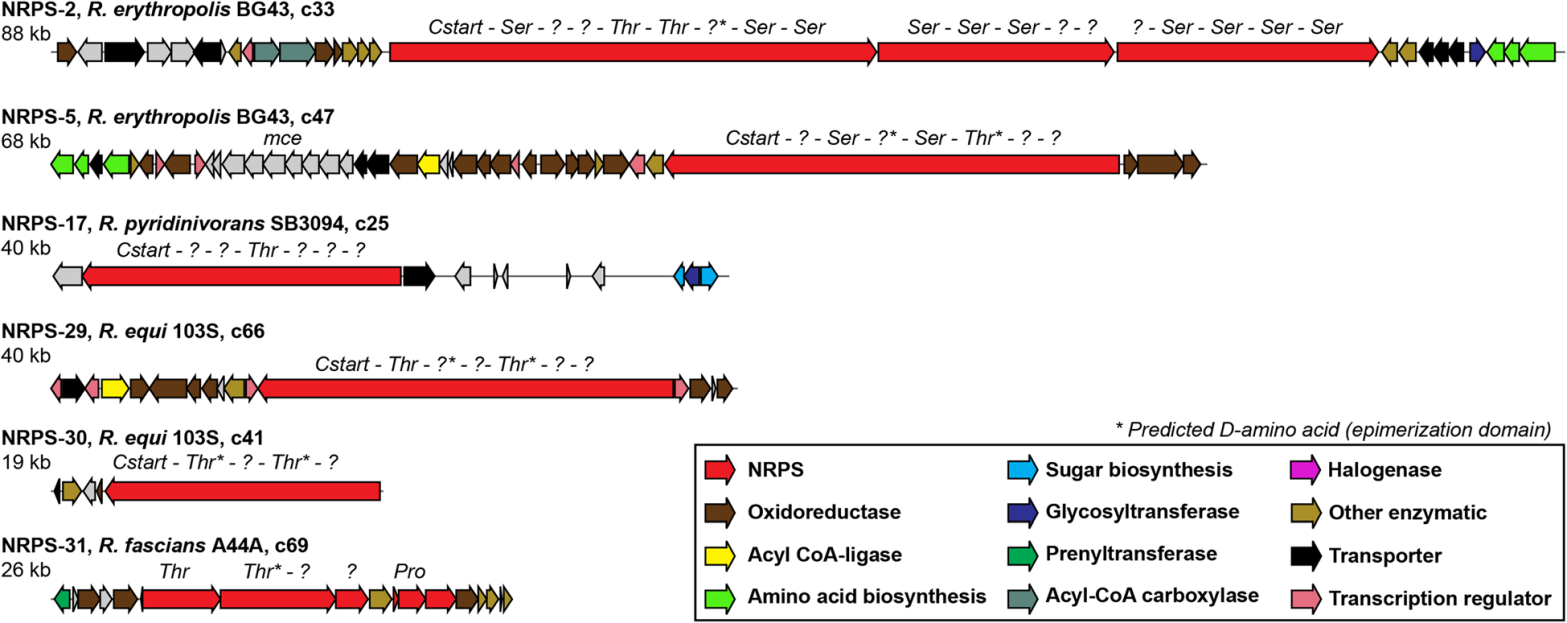
Nonribosomal-peptide BGCs that are potentially interesting for further studies. The amino acid residues predicted to be selected by the encoded NRPSs are indicated on top of each corresponding gene, and question marks indicate unknown substrates. The clusters shown are the following: NRPS-2, unknown and conserved in most rhodococci; NRPS-5, which contains an NRPS architecture similar to the humimycin NRPS; NRPS-17, a putative lipopeptide duplicated in the genome of *R. pyridinovorans* and present also in *R. equi* strains; NRPS-29 and 30, only present in *R. equi* strains; NRPS-31, a cluster believed to be involved in pathogenicity in *R. fascians*

Intriguingly, the genome of *Rhodococcus pyridinovorans* SB3094, which is the strain with the smallest genome of all free-living rhodococci studied in this work (5.24 Mb), shows a genomic duplication of a 366-kb region of its chromosome covering four of its BGCs which have not been characterized. The genus *Rhodococcus* is known to contain a great redundancy of genes but duplication of complete gene clusters has not been reported before [49]. Notably, in some industrial strains, copy number variation of BGCs has been shown to lead to increased specialized metabolite production [50]. A similar high-producing phenotype may have driven this evolutionary event and its fixation in a population; further experiments are required to confirm this. The combination of a small genome and duplication of 7% of it indicates that this strain is highly specialized to its environment [34, 51]. This strain was isolated from oil fields. In fact, the only NRPS cluster duplicated (**NRPS-17,** see Fig. 4), which is only shared with *R. equi* strains, has a C-starter domain, which is known to acylate the first residue of the NRP [15], indicating that it probably encodes the biosynthesis of a lipopeptide that could potentially serve as biosurfactant. Surfactants are compounds that decrease the surface tension of two fluids and improve the availability of hydrophobic compounds such as oil, which is probably key to the survival of this strain. Interstingly, *R. pyridinovorans* is also the only strain along with *Rhodococcus sp.* AD45 to lack a butyrolactone BGC. This cluster is known to be very conserved in *Rhodococcus* and is thought to play an important role in this genus [12]; in streptomycetes, the γ-butyrolactone molecules are known to be involved in the regulation of their specialized metabolism.

### NRPS clusters constitute the most dominant biosynthetic class in rhodococci

**Fig. 5:**
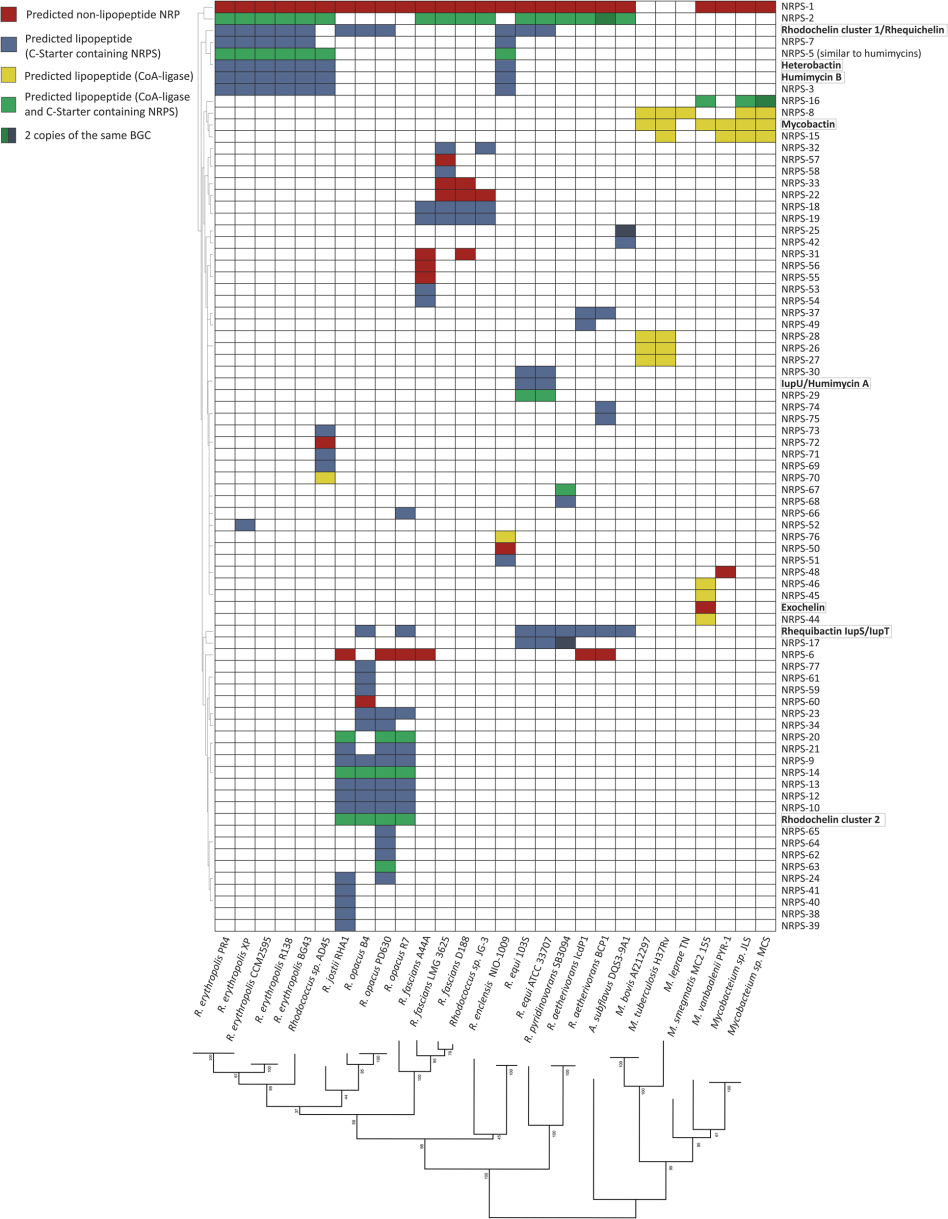
Presence/absence map showing all NRPS clusters in the different strains. White indicates absence of the cluster. Blue indicates presence of a gene cluster containing a C-starter domain in at least one of the NRPSs, which indicates that it encodes the biosynthetic pathway for a lipopeptide. Yellow indicates presence of a NRPS cluster containing an acyl CoA-ligase/synthetase, also indicative of the final product being a lipopeptide. Green indicates presence of NRPS clusters containing both one or more acyl CoA ligases/synthetases and a C-starter domain in one of the NRPS. Red indicates presence of NRPS clusters not predicted to synthesize lipopeptides

NRPS clusters are highly represented in *Rhodococcus* genomes. NRPSs can synthesize a great variety of peptides; more than 500 different precursors have been identified that can be used by NRPSs, thus creating a highly-varied array of compounds [16]. Each precursor amino acid added is specified by the adenylation domain of an NRPS module. Apart from this diversity of precursors, the peptide can be modified after it is released from the NRPS by other tailoring enzymes that produce significant changes in the structure. In total, 79 distinctive NRPS GCFs were found across all 28 strains. Only one of them is shared with all strains, except for the pathogenic *Mycobacterium* strains; the product of this shared cluster is not known. Most of the NRPS clusters are present only in one strain or only in a small group of related strains as is the case of the *R. erythropolis* clade*,* a clade comprising *R. opacus* strains and *R. jostii*, and the *R. fascians* clade (Fig. 5). In the case of *R. fascians*, it is possible that these clusters are involved in pathogenicity, as is believed to be the case for NRPS-31 (see Fig. 4) which was described in *R. fascians* D188 [52]. Mutagenesis and expression studies performed with this gene cluster revealed that it plays a role in pathogenicity but is not essential [52]. This cluster is located on the plasmid of this strain, but the final product of the cluster and its physiological role are still unknown. Interestingly, some taxonomically distantly related species share BGCs: for example, the genome of *Rhodococcus enclensis* NIO-1009 contains five NRPS BGCs (including the one for humimycin B) that are otherwise only found in genomes belonging to the *Rhodococcus erythropolis* group. Given the fact that these BGCs are located in close proximity, a large-scale horizontal gene transfer event might explain this. As *R. enclensis* and *R. erythropolis* are found in similar habitats (soils, sediments), this could be another example of how, sometimes, BGC repertoires are shared within the same ecosystem, and thereby correlate stronger with ecology than with taxonomy [53].

A few NRPS gene clusters that have been described in different strains of *Rhodococcus* represent siderophores in all cases, reflecting the importance of iron uptake for these bacteria. The importance of iron in rhodococcal physiology (and, arguably, in actinobacterial physiology in general) is also corroborated by the different gene clusters detected containing genes encoding the biosynthesis of heme groups, porphyrin and iron-sulphur cofactors. The gene cluster involved in the synthesis of one important iron-scavenging molecule, the hydroxamate-type siderophore rhodochelin, has been described in *R. jostii* RHA1 [17]. This cluster was predicted in most *Rhodococcus* strains. The corresponding gene cluster from *R. equi* 103S was identified previously as responsible for the synthesis of rhequichelin [18]. The encoded protein sequences and gene order in the clusters are very similar, and therefore they are grouped together in our network. Some of the genes present in each cluster vary between species and as described by Miranda-Casaluengo et al. [18], three out of four adenylation domains from the NRPSs are highly conserved between the species with the only differences being two active site residues in the second adenylation domain [18]. Another hydroxamate-type siderophore in this case only present in *R. erythropolis* strains, *R. enclensis* and in *R. sp.* AD45, is heterobactin, described in *R. erythropolis* PR4 by Bosello and collaborators [19]; this is grouped together with **Rhequibactin IupS/IupT** in the network and has a structure similar to rhodochelin (Additional file 1: Figure S3).

In the case of rhodochelin, further genes are involved in its biosynthesis, located outside this gene cluster [17]. This NRPS gene cluster thus is a good example of how very similar NRPS clusters can be responsible for the production of different molecules. Another group of NRPS-synthesized siderophores described from *Rhodococcus* is constituted by the catecholate-type siderophore rhequibactins synthesized by the rhequibactin BGC [20, 54]. These siderophores were described in *R. equi* 103S and two different NRPSs are involved in its biosynthesis, IupS and IupT; it is thought to be used only in saprophytic growth, since its deletion did not affect the pathogenicity but prevents the growth of the strain as a free-living organism [20]. The *iupU* gene was also described in this strain by Miranda-Casaluengo et al. [20] and was believed to be related to rhequibactin biosynthesis. The molecular product of this pathway was predicted to be a non-soluble siderophore believed to have a function similar to mycobactin, which is cell wall-bound and therefore not diffusible [36]. Recent work, describing the *Rhodococcus* antibiotics humimycins, identified the *iupU* gene as encoding the NRPS responsible for the synthesis of the molecule humimycin A [26]. Humimycins were synthesized based on the predicted product of the NRPS encoded by IupU and by another NRPS present in the *R. erythropolis* genome (encoding the biosynthesis of a variant, humimycin B). Both molecules showed potent activity against methicillin-resistant *Staphylococcus aureus* (MRSA), by targeting lipid II flippases [26]. The NRPSs contain a C-starter domain, which indicated that the first residue of the NRP is acylated. Therefore, a β-hydroxymyristic acid was added to the N-terminal residue of the compounds, but no further modification was done in the product. It is not unthinkable that the actual natural products are further modified by other enzymes encoded in these BGCs (they contain a conserved amidohydrolase, an N-Acyltransferase and a MbtH protein, and sometimes a dehydrogenase). While Miranda-Casaluengo et al. [20] show that the expression of *iupU* is not controlled by iron, as is normally the case for siderophores, its deletion does affect growth of *R. equi* 103 s in low iron conditions when growing as a free-living organism. Of course, it remains very possible that IupU does synthesize a real, humimycin-like antibiotic: for example, one or more intermediates of the synthesis could be affecting the expression of rhequibactin, which would provide an alternative explanation for the low-iron phenotype of the knockout. Interestingly, yet another NRPS GCF, **NRPS-5** (see Fig. 4), which is present in all *R. erythropolis* strains, has an NRPS with the same domain architecture as the humimycin NRPSs, but with different predicted substrates in modules 1, 3, 4, 6 and 7. This cluster also contains a long chain fatty acid CoA-ligase-encoding gene, as well as a mammalian cell entry (*mce*) operon. Further experimental studies will be needed to verify the natural physiological roles of each of these intriguing nonribosomal peptides.

Two mycobacterial GCFs encoding siderophore-producing NRPSs were detected in our analysis, which are responsible for the production of the hydroxamate-type siderophore **mycobactin** and **exochelin** [36], respectively. The second set of genes necessary for mycobactin biosynthesis, located on a different locus, were only detected in *M. tuberculosis* [55]. Mycobactin is known to be essential for *M. tuberculosis* pathogenicity. It is hydrophobic and is localized in the cell wall, and is thought to work together with the siderophore carboxymycobactin, which is a soluble siderophore [36]. It is believed that carboxymycobactin transfers the iron to mycobactin, which is then reduced from Fe^3+^ to Fe^2+^ and transported into the cytosol [36]. The transfer is thought to be mediated by the iron-dependent membrane protein HupB [36]. The mycobactin BGC was detected in every *Mycobacterium* strain except for *M. leprae* TN. Exochelin has been described in *M. smegmatis* [56]. In the genome annotations that we used, the NRPS is encoded in only one ORF instead of two. FxbB and FxbC are fused and the BGC containing this fused NRPS was only detected in *M. smegmatis*.

The products of the other NRPS GCFs (71 in total) remain unknown, which suggests a great potential for identifying novel nonribosomal peptides. The clusters **NRPS-29 and NRPS-30** (see Fig. 4) are only present in the facultative horse pathogen *R. equi*. This strain has also been described as a human opportunistic pathogen with a pathogenicity mechanism similar to that of *M. tuberculosis,* mainly infecting alveolar macrophages; this has attracted large interest towards the strain [57]. Mutational inactivation of these gene clusters, followed by tests on infective abilities and survival of these mutants in host cells, should clarify whether they can be used to develop drugs that may target any of these enzymes, or even a vaccine.

### Putative lipopeptides

We observed that several of these NRPS gene clusters contain individual fatty acid CoA-ligases/synthetases as well as C-starter domains [15], suggesting that their final products may be lipopeptides. Many of these peptides are predicted to be rich in serine and threonine residues (see examples in Fig. 4), although these predictions may not be reliable given the distant relationship to any adenylation domains with known specificity. *Rhodococcus* strains seem to have a preference for lipopeptides formed by a C-starter domain or the combination of C-starter domain and an acyl CoA-ligase/synthetase, while *Mycobacterium* strains have a bigger proportion of clusters with a CoA-ligase and without C-starter domains (Fig. 5). Lipopeptides include antibiotics, such as daptomycin, the last major antibiotic that has been commercialized [58]. However, lipopeptides have other functions besides antibiotics: some function as surfactants, others display haemolytic activity [59] and still others play a role in establishing infection and/or biofilm formation [60]. They are formed from a cyclic oligopeptide, non-ribosomally synthesized, to which an acyl chain is attached [61]. These compounds are known to have different antimicrobial activity and toxicity depending on the length of the acyl chain. The acyl chain can be attached to the oligopeptide by different methods [62]: through a stand-alone acyl-carrier protein (ACP) and fatty acid ligase, as is the case of daptomycin, by a hybrid NRPS/PKS enzyme containing an ACP and fatty acid ligase domain as is the case for mycosubtilin, or by a specialized C-starter domain in the NRPS, as is the case for surfactin. In the case of the calcium-dependent antibiotic from *Streptomyces* species, the fatty acid is synthesized in a specific pathway (Fab enzymes encoded in the CDA gene cluster and enzymes from the primary metabolism), and is then attached to a stand-alone ACP that directly transfers the lipid to the condensation domain of the NRPS where it is attached to the peptide [63]. Of the 79 distinct NRPS GCFs found our analysis, 69 show hallmarks of encoding the biosynthesis of lipopeptides. Also, all rhodococcal genomes studied encode putative lipopeptide BGCs, regardless of their specific ecological diversity. Still, the wide variety of bioactivities known to be associated with lipopeptides explains why they are likely to be important in various niches: They may aid the infection process in pathogenic strains or function as antibiotics in saprophytic ones. To zoom in on the biosynthetic diversity of lipopeptides encoded in rhodococcal genomes, we performed a detailed analysis of the fatty acid CoA-ligases and synthetases encoded in NRPS clusters. Out of the 79 GCFs, 19 encoded distinct CoA-ligases that may be involved in lipopeptide biosynthesis [13, 64]. Notably, such ligases may also be encoded outside the cluster: for example, the enzymes in charge of the transfer of the acyl chain to the peptide part of mycobactin are in different loci as the peptide synthetases [55]. A phylogenetic study of these enzymes, comparing them to previously described fatty acid CoA-ligases involved in the synthesis of characterized lipopeptides (Additional file 1: Figure S4), highlights their diversity.

Given the fact that such a wide variety of rhododoccal lipopeptide BGCs exists, we hypothesize that they have adapted to specific ecological sub-functions during evolution. If this indeed is the case, we predict that a dynamic evolution of lipopeptide BGC repertoires has occurred. In order to study this dynamic evolution, we performed ancestral state reconstruction of the 67 clusters using the software Count [37] to identify GCF gain/loss events across the evolutionary history of the *Rhodococcus* genus (Additional file 1: Figure S5). Indeed, the vast majority of putative lipopeptide GCFs showed a taxon-specific distribution. None are conserved throughout *Mycobacterium* and *Rhodococcus*, but **NRPS-2** and **rhequibactin IupS/IupT** are present in several strains of *Rhodococcus* and in *A. subflavus* (Figs. 3 and 4). The clade with the largest number of predicted lipopeptides is the one containing *R. jostii* RHA1 and the three *R. opacus* strains. With 17 putative lipopeptide BGCs, *R. opacus* P630 contains the largest number, followed by *R. jostii* RHA1 with 15 putative lipopeptide BGCs. The other *Rhodococcus* clades harbour between three and eight putative lipopeptide BGCs per genome. The *R. jostii* RHA1 and *R. opacus* PD630 and B4 strains have been studied for their ability to accumulate carbon in triacyclglycerols (TAGs) when growing in nitrogen limited media and using different carbon sources [49, 65, 66]. Alvarez et al. [65] have shown a higher triacyclglycerol accumulation when using gluconic acid as the sole carbon source and an even higher production in the case of strain PD630 when grown in olive oil [49, 65]. Lipopeptide surfactants might facilitate degradation of the hydrophobic compounds present in their growth media, allowing their import into the TAG biosynthesis pathway. Interestingly, *M. tuberculosis* has five putative lipopeptide GCFs (**NRPS-15, NRPS-26, NRPS-27, NRPS-28 and NRPS-8**), in addition to mycobactin, making it the *Mycobacterium* strain with the largest number of lipopeptides; it should be noted though, that all of these lipopeptides are predicted as such based on the presence of a CoA-ligase-encoding genes, which could also have a different enzymological role (and hence is a ‘weaker’ marker for peptide lipidation then C-starter domains are). Also, **NRPS-26** and **NRPS-28** are not canonical NRPS clusters; they do not possess a modular NRPS enzyme but stand-alone domains usually found in modular enzymes, as described in Wang et al. [67]. Lipopeptides are known to induce a strong immune response and many of them have remained uncharacterized [68, 69]. The products of these putative lipopeptide BGCs might allow development of vaccines against *M. tuberculosis* by inducing a response of the host immune system.

### Many rhodococci and mycobacteria contain strain-specific GCFs

**Fig. 6:**
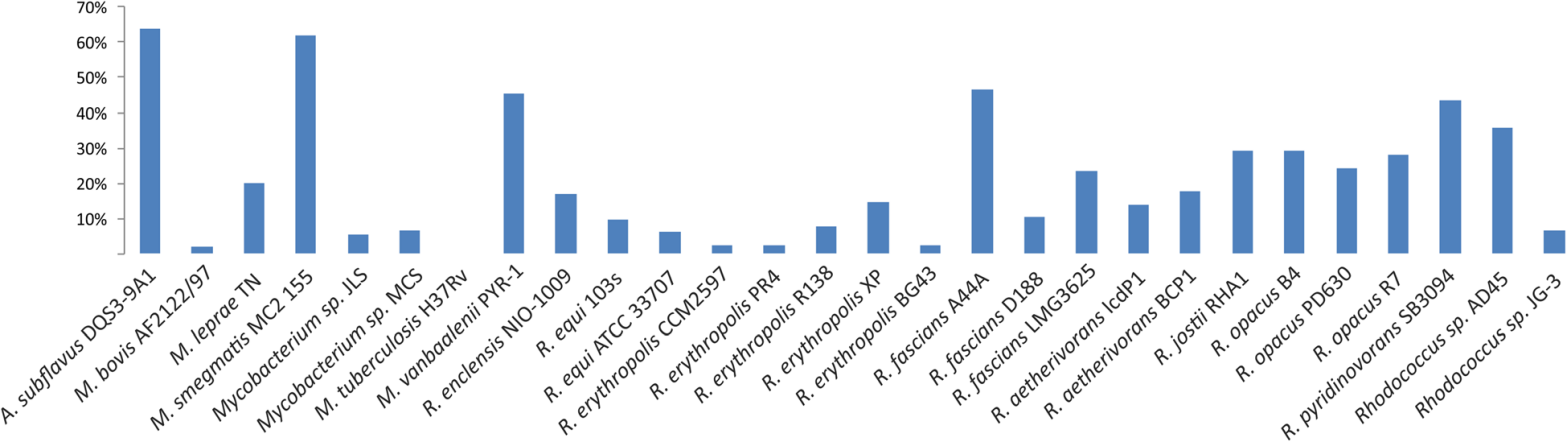
Percentage of strain-specific gene clusters from all detected GCFs present in each strain. *M. tuberculosis* is the only one that shares all its BGCs with other species, while *M. smegmatis* and *A. subflavus* only share 38.1% and 36.2% of their BGCs with the other strains studied. The number of gene clusters not shared by *R. fascians* A44A (46.4%) compared to its close relatives *R. fascians* LMG2536 (23.6%) and *R. fascians* D188 (10.3) is also striking but explained by their presence in different clades in the phylogenetic tree (Additional file 1: Figure S1), also observed in [70]

Every strain except for *M. tuberculosis* has strain-specific GCFs (Fig. 6). *M. tuberculosis* shares all its GFCs with at least one other strain, in many cases with *M. bovis*. The *M. smegmatis* and *A. subflavus* strains display the highest number of strain-specific GCFs (about 65%) followed by *M. vanbaalenii* and *R. fascians* A44. A. The number of shared BGCs will depend, of course, on how closely related one species is to the rest of the studied strains. *A. subflavus* is the only strain of its genus studied, therefore it was expected that it would have many strain-specific GCFs. On the contrary, this was not expected for the *R. fascians* strains. *R. fascians* A44A has almost 50% of strain-specific clusters, while only 10% and 23% of the GCFs from *R. fascians* D188 and *R. fascians* LMG 2536, respectively, are species-specific. Creason et al. previously indicated that *R. fascians* A44A is relatively distantly related to the other *R. fascians* strains [70]. Possibly, these differences are also responsible for host range specialization among members of this phytopathogenic species, as well as differences in the symptoms caused.

Another interesting observation is that most GCFs present on plasmids are unique to the species they are found in. This is true in the cases of *R. jostii* RHA1, *R. erythropolis* R138, all three strains of *R. opacus* and *R. pyridinovorans* SB3094. Plasmids are mobile and therefore the BGCs encoded on them might be expected to have homologues in more than one species. To find out whether these clusters are shared with other strains, which also may indicate which strains share the same habitat, *R. jostii* RHA1 was studied in more detail. Fifteen out of the 35 non-shared clusters were found to be present on the plasmids of *R. jostii* RHA1. This species has a total of 18 clusters on its three plasmids, thus most of them are not shared with the rest of the species in this study, not even with its closest relative *R. opacus*. The number of strain-specific clusters present on the plasmids of *R. jostii* RHA1 is comparable to that in *R. opacus* R7, which has 11 strain-specific clusters on its plasmids from 37 non-shared of a total of 128 predicted gene clusters. This number is much higher than the number of strain-specific clusters present on the plasmids of their close relatives *R. opacus* PD680 and *R. opacus* B4: only three strain-specific clusters are present on the plasmids of *R. opacus* PD680 and five in *R. opacus* B4. Due to the previously mentioned special nature of the *R. pyridinovorans* SB3094 genome, which is small and has a 366 kb duplication, we also analyzed the two BGCs predicted on its plasmid. Homologs of the enzyme-coding genes in both these clusters were also found in other rhodococci isolated from oil-contaminated soil, soil, waste water, and even in *Rhodococcus gordonia*, which has been isolated from clinical material and phenol-contaminated soil. This suggests that indeed these BGCs may confer specific traits to the strain that allow it to thrive in such conditions. Experimental studies are needed to further characterize these clusters.

### Activation of cryptic BGCs may allow discovery of new bioactive compounds

As presented in this work, most BGCs identified in the different strains studied here are completely unknown. Various methods have been developed to induce the expression of such BGCs. Some of these approaches are the deletion of known biosynthetic pathways to make precursors available for other routes [71], the co-cultivation of two or more strains [72], the manipulation of regulatory systems [73, 74] or the heterologous expression in other strains [75]. In some cases, introduction of synthetic promoters may be needed to enforce BGC expression in the heterologous host [76]. These and other synthetic biology techniques can be used for the activation of cryptic metabolic routes; the final goal of synthetic biology in this context is to be able to design molecules by combining different regulatory elements and biosynthetic genes (building blocks) [77, 78]. As such, it has the potential to allow for the refactoring as well as further engineering of cryptic gene clusters such as those studied in this paper. Powered by such technologies, the secondary metabolism from *Rhodococcus* can hopefully be used to identify targets to fight related pathogenic strains as well as to identify and engineer novel bioactive natural products.

## Conclusions

Rhodococci harbour a great number of putative biosynthetic gene clusters in their genome, most of them of unknown product. These include a considerable number of biosynthetic gene clusters that putatively encode the production of lipopeptides, which can have diverse functions, ranging from biosurfactants to antibiotics. *Rhodococcus* strains also share several BGCs of unknown function with free-living and pathogenic strains of *Mycobacterium*. This genus is therefore interesting to explore for finding new natural products as well as finding conserved pathways that could be used as targets to fight pathogenic strains.

## Methods

### Software

Links and references to the software used in this study can be found in Additional file 4.

### AntiSMASH analysis

Genome sequences were obtained from NCBI (http://www.ncbi.nlm.nih.gov) and analysed with antiSMASH, which was run with default settings plus inclusion of the ClusterFinder algorithm (http://antiSMASH.secondarymetabolites.org). Results can be accessed in Additional file 5.

### 16S rRNA phylogenetic tree

16S ribosomal RNA sequences were obtained from NCBI. In the case of species that contain more than one 16S rRNA genes, only one of them was included in the phylogenetic analysis since all of them appeared together in the tree. The analysis was performed which was performed using MEGA 6.0 [79] (http://www.megasoftware.net/). Multiple-sequence alignment was performed using MUSCLE with default parameters; a phylogenetic tree was generated using the Neighbour-Joining method and the bootstrap test with 1000 replicates.

### Phylogenetic tree of acyl-CoA ligases involved in the synthesis of lipopeptides

All acyl-CoA ligases present in all gene clusters detected by antiSMASH were identified by sequence homology (using the AMP-binding Pfam domain and BLAST queries against known CoA-ligases from MIBiG, see below). Acyl-CoA ligases not present in NRPS gene clusters and the ones corresponding to NRPSs adenylation domains were discarded for the analysis. A set of known acyl-CoA ligases involved in synthesis of lipopeptides were obtained from the MiBIG database and added to the analysis. Protein sequence alignment was performed using PROMALS3D. Phylogenetic analysis was performed using MEGA 6.0 the Neighbour-Joining method and the bootstrap test with 1000 replicates.

### BGC similarity networks and gene cluster family reconstruction

A network containing 2363 nodes was generated using BiG-SCAPE (https://git.wageningenur.nl/medema-group/BiG-SCAPE), with parameters optimized for a good separation of NRPS clusters (see Additional file 3). The parameters used were Jaccard index (0.2), similarity of domain order measured by the Goodman-Kruskal γ index (0.05) and the domain duplication similarity, weighted by sequence identity (0.75). Six network versions were produced with different cut-offs of 0.60, 0.65, 0.70, 0.75, 0.80 and 0.85. The lower the cut-off, the fewer connections are kept between the clusters. Based on manual inspection of connections to known BGCs at various versions of the network, a cut-off of 0.75 was chosen for the final analysis.

### Analysis of the network

Groups of gene clusters with more than 7 nodes were curated manually based on inspection of the results of multi-gene homology searches with MultiGeneBlast (http://multigeneblast.sourceforge.net/) to confirm that they indeed constitute a valid GCF. Gene clusters of known molecular class but unknown final product (NRPs, PKs, RiPPs, etc.) were considered as the same cluster when the identity of the main biosynthetic enzymes was 60% or higher inside the same genus and 50% from different genera, given the lower identity expected between distinct genera. If no core scaffold biosynthesis enzymes could be identified in BGCs within a connected component, the organization predicted in the network was considered correct. Known and described GCFs were also curated manually with MultiGeneBlast and added or removed when required. Additional file 2 contains an overview of curated GCFs.

### Gain/loss diagrams

The gain/loss diagram was created using Count [37]. A total of 112 GFCs (GCFs shared with more than 7 strains and all predicted NRPSs) were used as input. Dollo parsimony and Wagner parsimony (gain penalty: 1) were used for the analysis.

### Presence/absence map generation

The Python Module Seaborn was used to generate the presence/absence map of GCF absence/presence across genomes. Pairwise distances were calculated using the Euclidean method, the hierarchical clustering of the gene clusters was performed with the Complete method. Strains were ordered according to their appearance in the 16S–rRNA based phylogenetic tree. In cases of strains containing more than one 16S rRNA gene, they were checked for consistent signal and the first occurring 16S rRNA in each strain chromosome was used to generate the phylogenetic tree from the presence/absence map and the three *R. opacus* strains which have 44 to 45 BGCs.

## Additional files

Additional file 1: Supplementary information. (DOCX 4395 kb)

Additional file 2: Overview table of all GCFs. (XLSX 24 kb)

Additional file 3: BGC sequence similarity network. (ZIP 2775 kb)

Additional file 4: List of software used in this manuscript. (XLSX 12 kb)

Additional file 5: antiSMASH results of every strain discussed in this manuscript. (DOCX 28 kb)

## Abbreviations

ACP: Acyl carrier protein
BGC: Biosynthetic gene cluster
GCF: Gene cluster family
NRPS: Nonribosomal peptide synthetase
PCP: Peptide carrier protein
PKS: Polyketide synthase
