# Supplementary material for: Genome-based exploration of the specialized metabolic capacities of the genus Rhodococcus
Source: BMC Genomics. 2017 Aug 9;18:593. doi: 10.1186/s12864-017-3966-1 (PMC5550956; doi:10.1186/s12864-017-3966-1)
Supplement: Supplementary file 1 — Supplementary information. (DOCX 4395 kb) [file 12864_2017_3966_MOESM1_ESM.docx]

**Supplementary information 1**


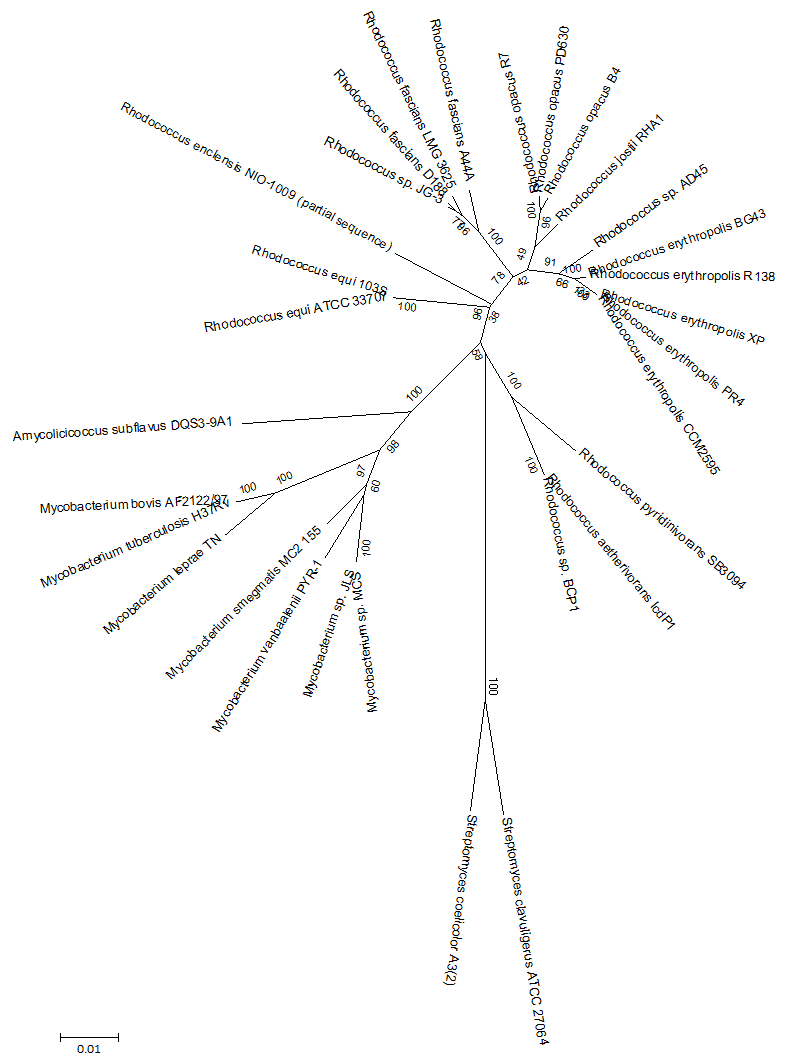


Figure S1. Neighbour-joining 16S rRNA phylogenetic tree of all 30 strains studied. A total of 1000 bootstrap replicates were performed in this analysis; bootstrap values are given in percentages.

**
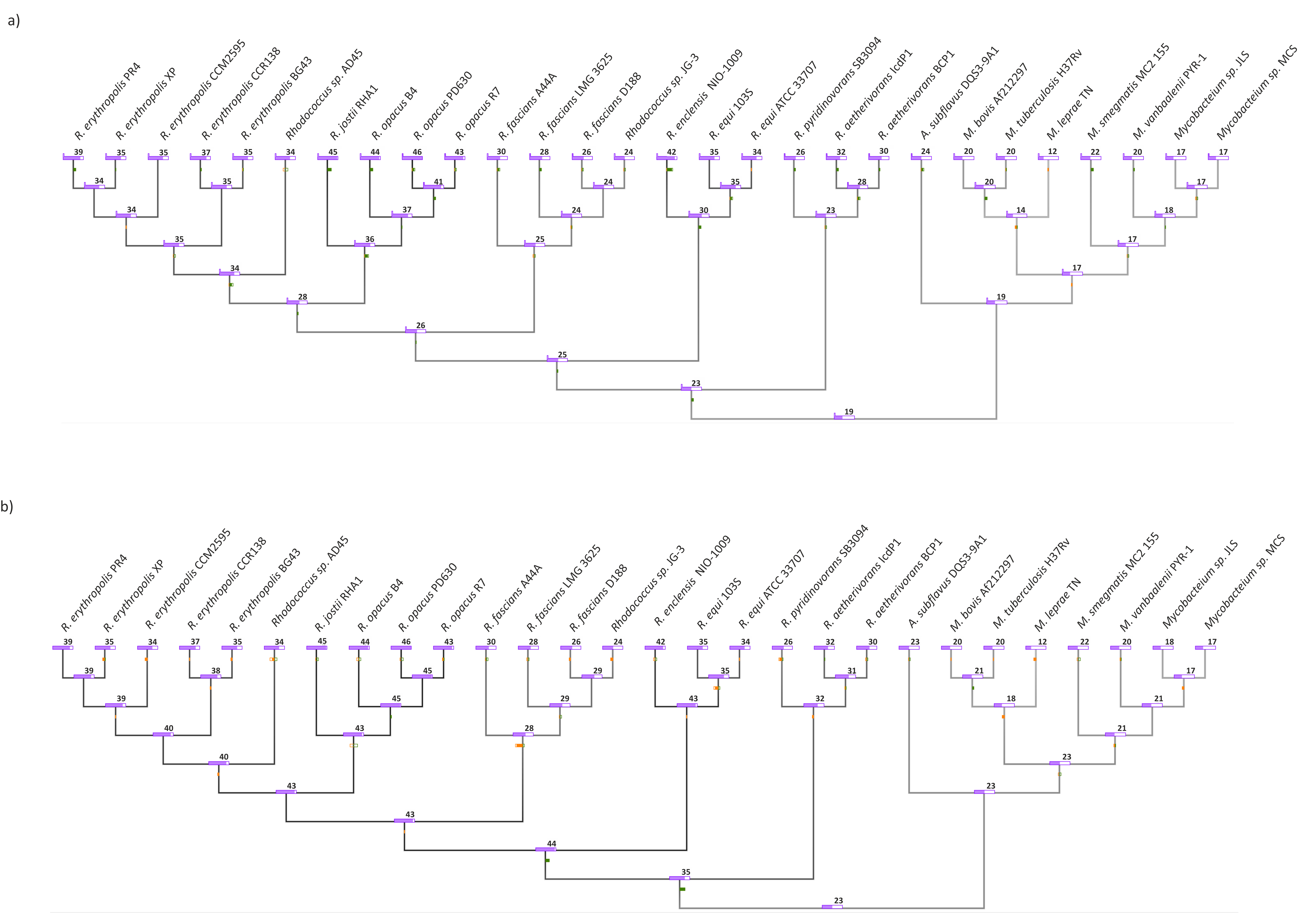
**

**Figure S2.** **Gain/loss diagram.** Clusters shared with more than 7 strains and all NRPS were analyzed for presence or absence in each strain and represented in the phylogenetic tree. Twenty-three GFCs are present in *Rhodococcus*, *Amycolicicoccus* *and Mycobacterium* strains (lower part of the dendogram). Horizontal lines under each node indicate the number of gained or lost clusters. Green to the right gain, yellow to the left loss. The purple bar on each node represents the proportion of clusters present in each strain from the total 113 clusters analyzed. a) Analysis made by Wagner parsimony with gain penalty=1. b) analysis made by Dollo parsimony.


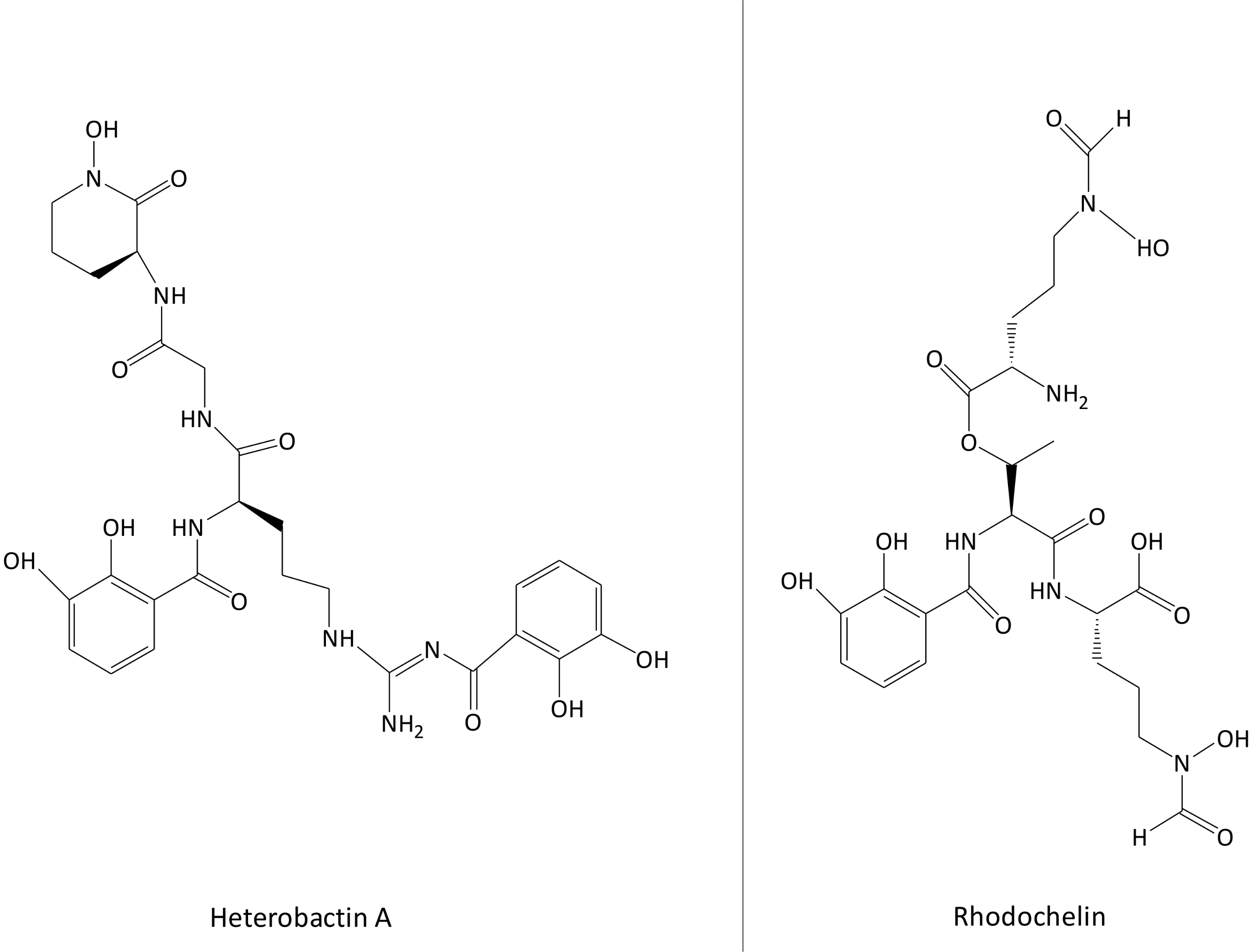


Figure S3. Structure of the siderophores heterobactin A and rhodochelin. Adapted from Bosello et al. [1, 2].


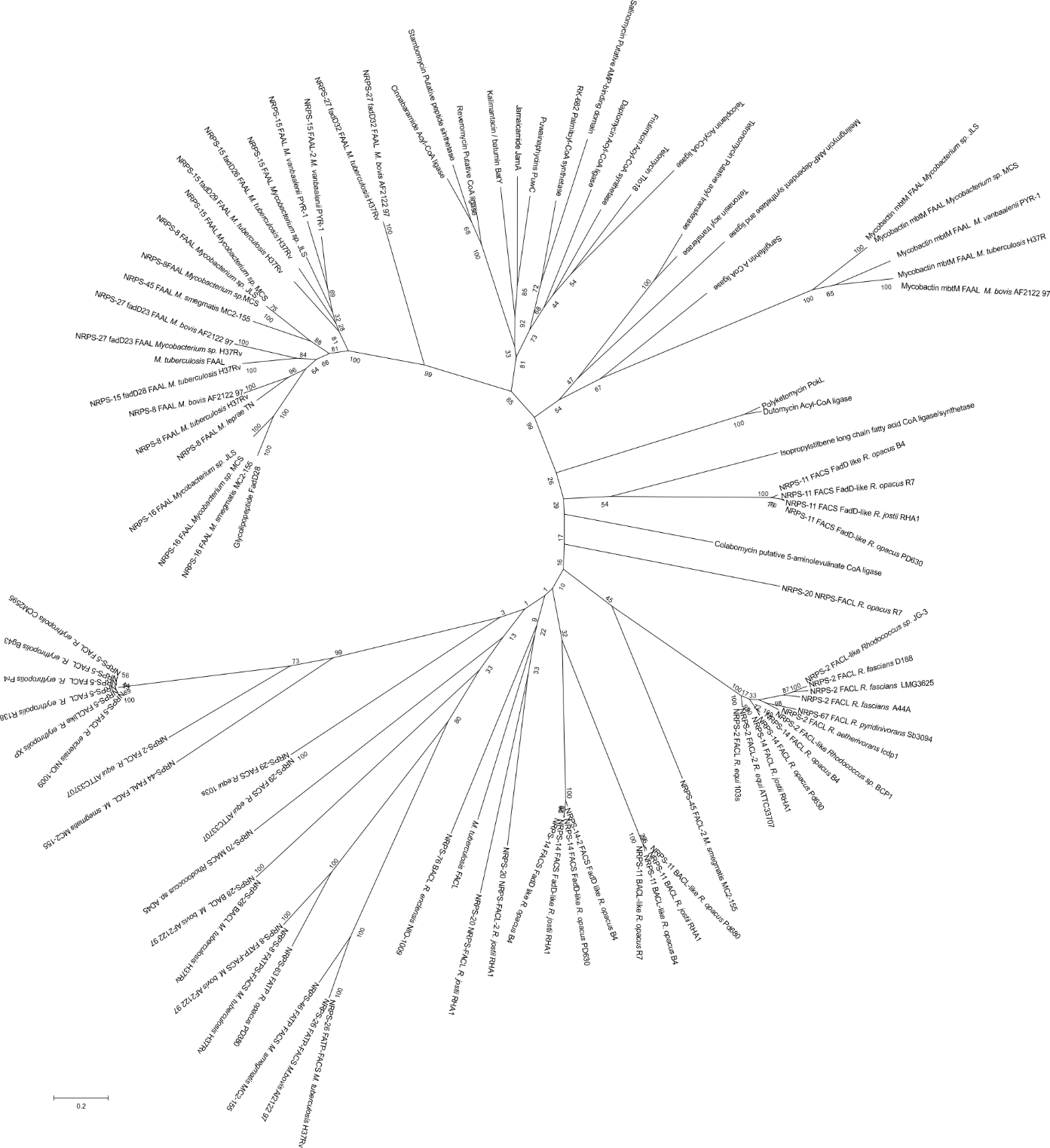


Figure S4. Phylogenetic analysis of the acyl CoA-ligases/synthetases detected in this study using experimentally characterized acyl CoA-ligases/synthetases as references. The Neighbour-joining method was used with 1000 bootstrap replicates. Bootstrap values are given as percentage. FAAL: Fatty acid AMP Ligases, FACS: Fatty acids Acyl-CoA Synthetases, FACL: Fatty acid Acyl-CoA ligases FATP: Fatty acid transport protein, MACS: medium-chain Acyl-CoA synthetase. BACL: Bile acid CoA ligases.


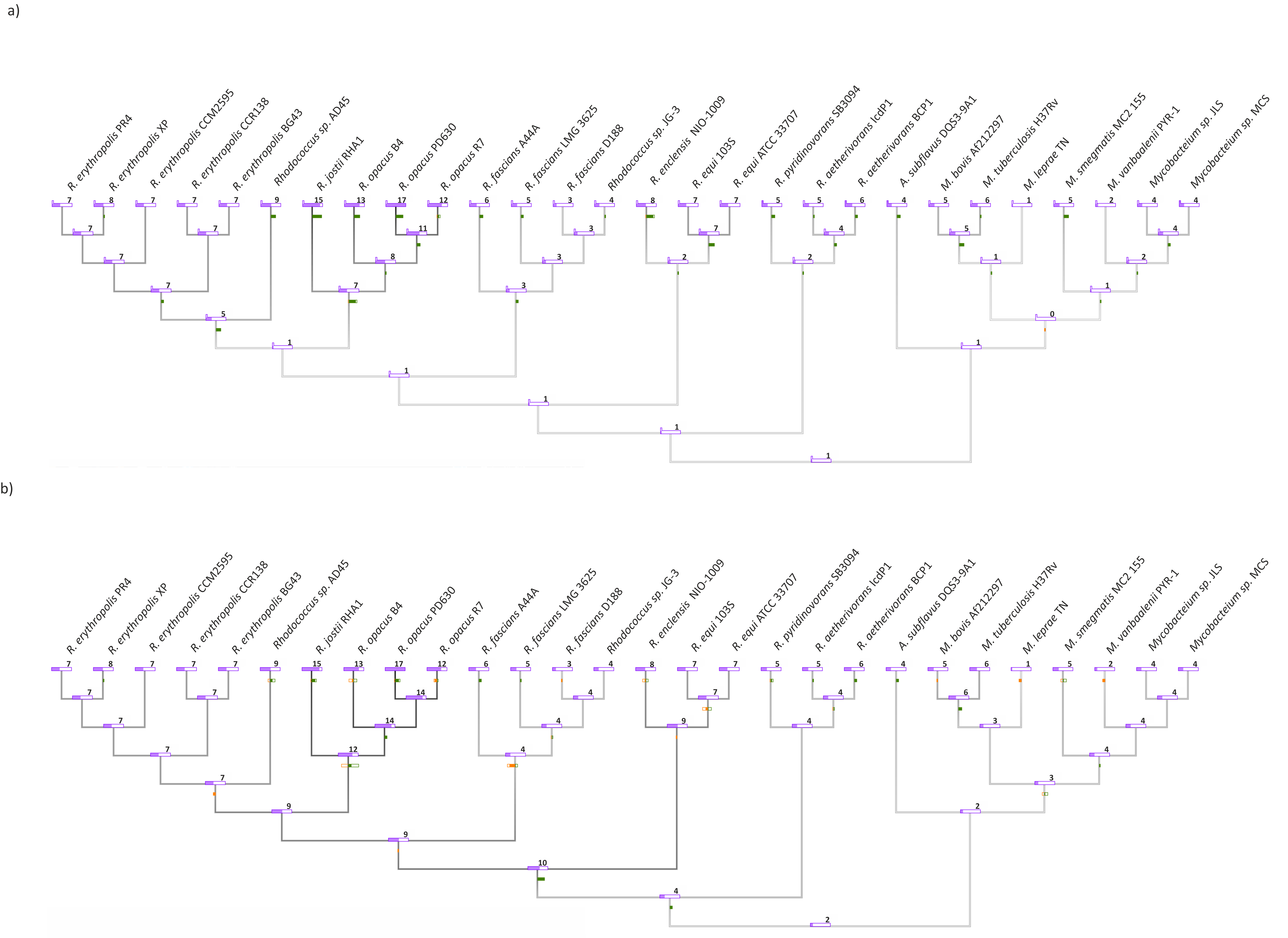


**Figure S5. Gain/loss events of the putative lipopeptides in all species**. Horizontal lines under each node indicate the number of gained or lost clusters. Green to the right gain, yellow to the left loss. The purple bar on each node represents the proportion of clusters present in each strain from the total 19 clusters analyzed. a) Wagner parsimony with a gain penalty of 1. b) Dollo parsimony

**Supplementary information 2**

**Description of Other-4, Other-5 and Other-6 BGCs.**

Other-5 is also present in *A. subflavus*. **Other-4** contains a homologue of a cutinase enzyme which in phytopathogenic organisms is involved in the infection process by degrading the plant cell-wall [47, 48]. These cutinase enzymes were also predicted in *M. tuberculosis* and are thought to be involved in providing substrates to form mycolic acids, or in pathogenicity [48]. Unfortunately, the function of the **Other-4** cluster could not be predicted in more detail. **Other-5** includes a protein with 92% identity to the IdeR global iron-dependent regulator described in *M. tuberculosis*, which is also homologous to the diphtheria toxin repressor DtxR from *Corynebacterium diphteriae* [49]*.* IdeR was also found in *R. equi* and *R. erythropolis* [50]. In pathogenic strains, it is known to regulate different virulence factors which are activated when the bacterial strain enters a host cell, where iron levels are scarce due to iron sequestering enzymes from the host as transferrin [49]. This cluster also includes an enzyme with a PAC2 (proteasome assembly chaperone) domain involved in the formation of the proteasome, which is essential for pathogenicity in *M. tuberculosis* [51]. A protein from the superfamily II of RNA and DNA helicases, a UDP-galactose-4-epimerase, two hydrolases and two hypothetical proteins are also encoded in this gene cluster. However, the products of the **Other-5** clusters remain unknown. Also for **Other-6** we could not identify a function. It encodes two multidrug transporters, which indicates that this cluster may be producing a bioactive compound. It also contains genes coding for three transcriptional regulators, a putative esterase, two dehydrogenases, a glutamate decarboxylase, a glutathione S-transferase, an acetyl transferase, a possible glycolate oxidase FAD-linked subunit, a possible enoyl-CoA hydratase, a monooxygenase and four hypothetical proteins.
