# Supplementary material for: Genome-based exploration of the specialized metabolic capacities of the genus Rhodococcus
Source: BMC Genomics. 2017 Aug 9;18:593. doi: 10.1186/s12864-017-3966-1 (PMC5550956; doi:10.1186/s12864-017-3966-1)
Supplement: Supplementary file 5 — antiSMASH results of every strain discussed in this manuscript. (DOCX 28 kb) [file 12864_2017_3966_MOESM5_ESM.docx]

<http://marnixmedema.nl/rhodococcus/Additional_file_5.zip>
